# Commentary: A channelopathy mutation in the voltage-sensor discloses contributions of a conserved phenylalanine to gating properties of Kv1.1 channels and ataxia

**DOI:** 10.3389/fncel.2018.00174

**Published:** 2018-06-20

**Authors:** Sonia Hasan, Therese Hunter, Gary Hunter, Mauro Pessia, Maria Cristina D'Adamo

**Affiliations:** ^1^Department of Physiology, Faculty of Medicine Kuwait University, Kuwait City, Kuwait; ^2^Department of Physiology and Biochemistry, Faculty of Medicine and Surgery University of Malta, Msida, Malta; ^3^Section of Physiology and Biochemistry, Department of Experimental Medicine, School of Medicine University of Perugia, Perugia, Italy

**Keywords:** KV1.1, channelopathy, potassium channels, voltage-gated, hydrophobic gating, shaker-related

In a recent article, we reported a novel heterozygous mutation in the *KCNA1* gene of a proband affected by episodic ataxia type 1 (EA1) (Hasan et al., 2017). The resulting substitution of a highly conserved phenylalanine in the Kv1.1 channel with a valine (p.F303V) led to positive shifts of voltage-dependence, altered kinetics of activation, deactivation and slow inactivation, reduced window currents, and decreased Kv1.1 current amplitude. Even with the critical location, within the voltage-sensing domain and amidst S4's renowned arginine-lysine motif, it remains intriguing that the mutation causing debilitating symptoms and a remarkable impact on gating involves the neutral non-polar phenylalanine.

In the open-state, the mobile S4 helix is stabilized by salt bridges formed between the charged residues that it contains and the acidic residues on S1 and S2 (Long et al., 2005). We constructed a model using rat Kv1.2 coordinates (Figure 1) that shows the open structure may further be stabilized by hydrophobic interactions. Substitution of the aromatic phenylalanine with the smaller aliphatic valine, as it appears in the mutation, resulted in weaker hydrophobic interactions between S4's F303V and both L339 and I335 of the S5 helix of a neighboring subunit. The mutation appears to reduce or abolish the effectiveness of these interactions, as well as that between side chains of F300 and F303 which lie in a stacked arrangement in the S4 helix, thereby destabilizing the channel's open state.

**Figure 1:**
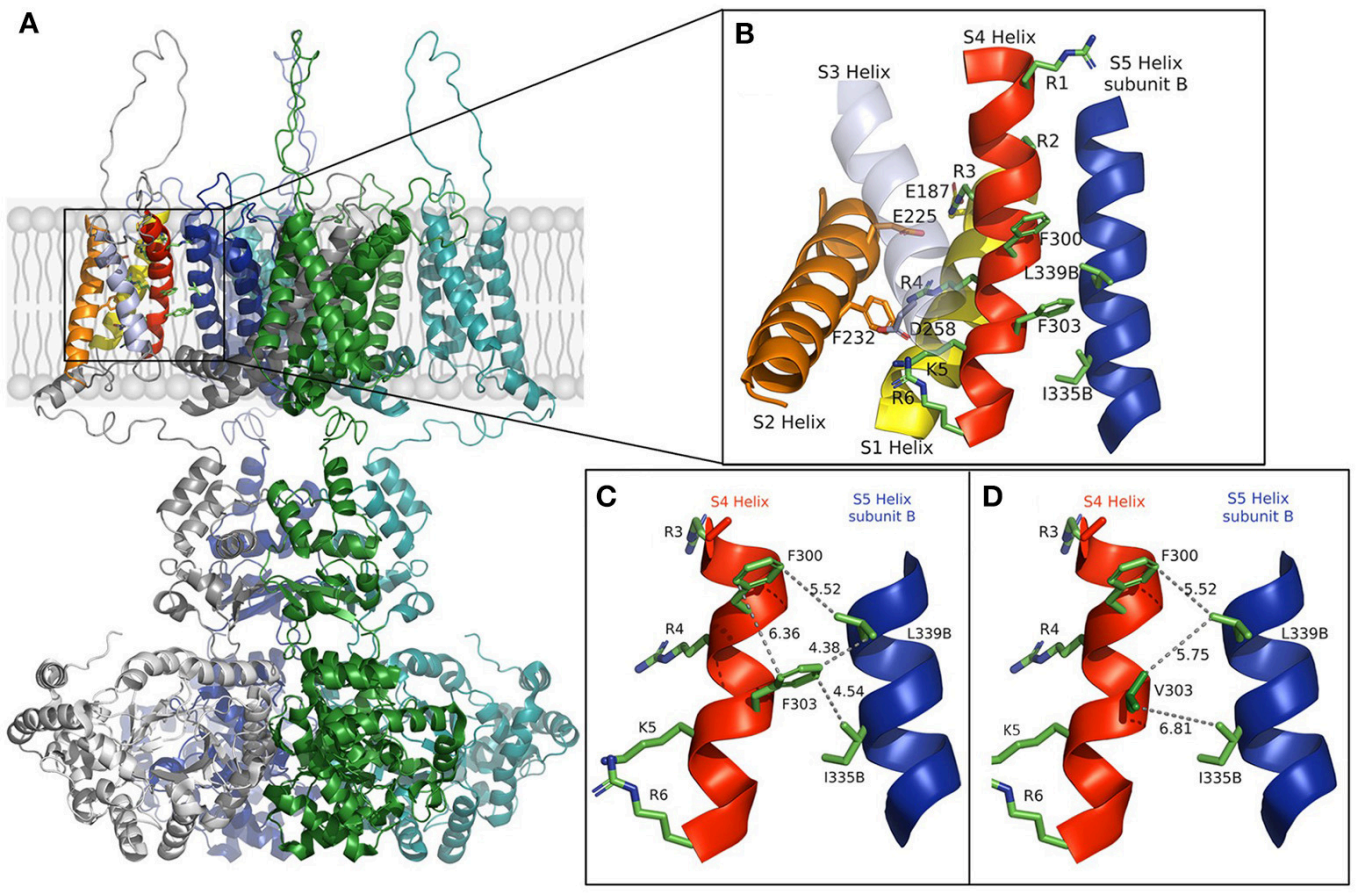
**(A)** The full model of the Kv1.1 potassium channel based on Kv1.2 coordinates is shown in the open state with subunits colored gray, green, blue, and cyan. **(B)** Enlargement of the section boxed in **(A)** with functional helices colored (S5, blue, is from a different subunit and labeled **B**). Residues involved in voltage sensing are drawn and labeled with those involved in the hydrophobic interactions between subunits. **(C,D)** The S4 helix and S5 helix of subunit **(B)** in the wild-type and F303V mutant, respectively. Interatomic distances are shown as dashed lines with distances shown in Å. A slight discrepancy in distances may apply due to the mobile nature of the S4 segment. The figure was drawn using The PyMOL (Molecular Graphics System, Version 2.0 Schrödinger, LLC), and adapted from Hasan et al. (2017).

In support of our results, mutagenesis of *Shaker* channel pore residues L409 and I405, that correspond to Kv1.1 L339 and I335, resulted in similar positive shifts of voltage-dependence of activation, while mutagenesis of a large number of other pore amino acids had no effect on gating (Yifrach and MacKinnon, 2002). This underlines the significance of residues F303, L339, and I335 to gating. In Yifrach and MacKinnon's mutagenesis study the majority of pore mutations that did have gating effects resulted in *gain-of-function* negative shifts of voltage-dependence of activation. The resulting enhanced open-state led to their interpretation that it is easier to disrupt a closed conformation than an open one. Nevertheless, all disease causing mutations in *KCNA1* reported thus far led to Kv1.1 *loss-of-function* more likely, as presented in our model, due to a disrupted open state. That the F303V mutation energetically favors a closed channel state goes with the observed biophysical properties of the channel; the changes in gating kinetics, particularly the decelerated activation and accelerated deactivation, and with the remarkable *loss-of- function*.

Reports of conserved hydrophobic residues that underlie, at least in part, the gating of voltage-dependent channels form the basis of the “hydrophobic gating” hypothesis (Jensen et al., 2012; Aryal et al., 2015). In support of this hypothesis are the many biophysical studies showing altered channel gating with modifications in cavity hydrophobicity (Imbrici et al., 2009; Jensen et al., 2010). Molecular dynamic simulations depicted pore closure accompanied by dehydration of the hydrophobic cavity that leads to the collapse of the pore at negative potentials (Jensen et al., 2010). Interestingly, simulations showed the voltage sensor domain needs to be intact in order to prevent collapse of the pore. In the absence of the voltage sensor the transition to a closed conformation occurred within a few microseconds. An accelerated deactivation also occurred as a result of the F303V mutation. F303 amid the voltage sensor motif is more than likely part of the hydrophobic gate proposed.

Similar to our findings, mutations involving phenylalanine residues as far from the pore as S1 and S2 have shown pronounced effects on gating. Hydrophobic interactions involving S2 segment's phenylalanine residue at position 233 stabilizes the channel's open configuration (Tao et al., 2010; Lacroix and Bezanilla, 2011; Schwaiger et al., 2013). Mutations of this phenylalanine to various non-aromatic residues speeds up deactivation while substitution to a tryptophan stabilizes the open state. Even more peripheral, the EA1 mutation F184C located in S1 displayed a 20 mV positive shift in voltage-dependence (Adelman et al., 1995). This phenylalanine interacts directly with gating charges of S4 and the selectivity filter of a neighboring subunit (Petitjean et al., 2015). Adding on to the intrinsic gating defects, the F184C mutation sensitizes Kv1.1 channels to extracellular Zn^2+^ (Cusimano et al., 2004; Imbrici et al., 2007). Zn^2+^ slows down activation rates, speeds up deactivation rates, shifts the voltage-dependence to more depolarized potentials and affects N-type inactivation. In *Shaker* channels the presence of the aromatic residue F401 of the S5 segment serves to inherently stabilize the open channel (Kanevsky and Aldrich, 1999). As for the EA1 mutation F414C, the channel was found completely non-functional despite its successful delivery to the plasma membrane (Imbrici et al., 2008). These findings suggest the bulky hydrophobic phenylalanine occupies a sterically confined area that allows essential hydrophobic interactions with nearby residues and restricts conformational movements toward channel closure.

We propose the rigid phenylalanine at position 303 as an open-state conformation stabilizing residue and aromatic-dependent interactions as a mechanism for the fine-tuning of conformational equilibria in the Kv1.1 channel, the dysfunction of which underlies EA1. Gating is more than likely the co-operative effort of many hydrophobic residues. An extensive mutagenesis approach to identify these multiple hydrophobic residues one at a time throughout the channel, and then to investigate the concerted effect of different residue mutations at the same time, is warranted. Obtaining more channel structure images in high resolution would provide clarification for the involvement of hydrophobic residues in stabilizing the channel in different conformational states. Theoretical and mathematical investigations compliment structural and functional results, however a more comprehensive method that includes all approaches is needed to assess the role of hydrophobic connections in channels. Some EA1 patients show improved symptoms with antiepileptics and carbonic anhydrase inhibitors, however no medication has thus far been proven effective. Aromaticity and hydrophobicity of residues Tyr-652 and Phe-656, located in the S6 domain of hERG channels, have been shown to underlie the basis of drug interactions with structurally diverse compounds (Fernandez et al., 2004). Future research should be directed toward finding a precise therapy tailored around critical hydrophobic and aromatic residues in the Kv1 channel.

## Author contributions

SH wrote the article. MP and MCD provided critique, suggestions, and edited the paper. TH and GH provided the Kv channel model figure.

### Conflict of interest statement

The authors declare that the research was conducted in the absence of any commercial or financial relationships that could be construed as a potential conflict of interest.
